# Phenolic Content, Antioxidant Activity, Anti-Inflammatory Potential, and Acute Toxicity Study of *Thymus leptobotrys* Murb. Extracts

**DOI:** 10.1155/2020/8823209

**Published:** 2020-09-18

**Authors:** Asmaa Oubihi, Hanae Hosni, Issmail Nounah, Abdessamad Ettouil, Hicham Harhar, Katim Alaoui, Mohammed Ouhssine, Zineb Guessous

**Affiliations:** ^1^Laboratory of Agro-Physiology, Biotechnology, Environment and Quality, Faculty of Science, Ibn Tofail University, Kenitra, Morocco; ^2^Pharmacodynamy Research Team ERP, Laboratory of Pharmacology and Toxicology, Faculty of Medicine and Pharmacy, Mohammed V University, Rabat, Morocco; ^3^Laboratory of Chemistry and Organic and Bioorganic Synthesis, Faculty of Science, Geophysics, Natural Patrimony and Green Chemistry (GEOPAC) Research Center, University Mohammed V, Rabat, Morocco; ^4^Laboratory of Nanotechnology, Materials and Environment, Department of Chemistry, Faculty of Science, Mohammed V University in Rabat, BP. 1014, Rabat, Morocco

## Abstract

*Thymus leptobotrys* is a medicinal plant belonging to the Lamiaceae family, endemic in Morocco, and used in traditional medicine. The present work aims to study the phenolic compounds, the antioxidant activity, the anti-inflammatory effect, and the toxicity of two ethanolic and methanolic extracts of *Thymus leptobotrys* aerial part. The yield of the methanolic extraction (22.2%) is higher than that of the ethanolic extraction (15.8%) and is characterized by higher contents of polyphenols 243.08 mg/g GAE (mg/g of gallic acid), flavonoids 179.28 mg/g RE (mg/g of rutin), and tannins 39.31 mg/g CE (mg/g of catechin). The *in vitro* measurement of antioxidant activity with the 2,2-diphenyl-1-picrylhydrazyl (DPPH) radical reduction test and Trolox equivalent antioxidant capacity (TEAC) test demonstrates the higher performance of the methanolic extract. The evaluation of the anti-inflammatory effect *in vivo* on adult Wistar female rats leads to a very significant decrease in the inflammation of the edema compared to the standard drug (indomethacin) and the control group. The toxicity test reveals that both extracts showed no toxicity within an LD50 above 2000 mg/kg body weight of the rats.

## 1. Introduction

Oxidative stress causes significant damage that accelerates cellular aging. This aging process leads to serious pathologies such as cancer, cardiovascular disease, diabetes, neurodegenerative disease, inflammation, digestive diseases, and metabolic syndrome [1–3]. Several situations in daily life lead to the production of free radicals responsible for oxidative stress. These include endogenous overproduction of inflammatory oxidative agents, free radical overproduction (tobacco, alcohol, drugs, pollution, and pesticides), and a decrease in the antioxidant capacity of foodborne vitamins and trace elements [2, 4, 5]. The use of currently available synthetic antioxidant molecules is being questioned because of potential health risks [2, 6]. The search for natural antioxidants, mainly carotenoids, polyphenols (flavonoids and phenols acids), and vitamins (mainly vitamins C and E) [7] derived from plant materials, is growing rapidly and is based on the characterization of endemic plants. Typically, these natural antioxidants, particularly polyphenols, show evidence of a broad variety of biological effects, such as antiplasmodial, antimicrobial, antiarthritic, anticancer, and anti-inflammatory effects [8, 9].

The medicinal plant sector in Morocco has a rich and varied flora with very marked endemism [10]. The species of Thymus L. (Lamiaceae) are perennial, aromatic herbs, and widely used in the Mediterranean Basin [11]. *Thymus leptobotrys* Murb. is an endemic species of southern Morocco traditionally used in the treatment of bronchitis, indigestion, whooping cough, and rheumatism [12] and has antifungal, antimicrobial, analgesic, insecticidal, antioxidant, and antiviral properties [13–16].

No studies regarding the *in vivo* anti-inflammatory effect of *Thymus leptobotrys* were conducted previously to our knowledge. The present work is part of the valorization of Moroccan aromatic and medicinal plants. Its objective is to study the phenolic compounds, the antioxidant activity, the *in vivo* anti-inflammatory effect, and the acute toxicity of ethanolic and methanolic extracts of *Thymus leptobotrys*.

## 2. Materials and Methods

### 2.1. Plant Material

The aerial parts of *Thymus leptobotrys* are recovered in the region of Sidi Mzal, a small Berber village in the mountains of the Moroccan Anti-Atlas (N 29°86′/W 08°88′) during the harvest period (April–June 2017). The identification is made at the Laboratory of Botany and Plant Protection, Ibn Tofaïl University, Kenitra, Morocco. The sample is dried at room temperature and then ground and stored in sterile bags until analysis.

### 2.2. Animals

Adult female Wistar rats (180 to 230 g) are used for testing anti-inflammatory activity and acute toxicity. The animals are reared in the Animal Center of the Faculty of Medicine and Pharmacy of Mohammed V University in Rabat, under standard experimental conditions, where the temperature varies between 20 and 25°C with a 12-hour photoperiodic cycle. The study was conducted in accordance with the accepted principles set out in the report “Guide for the care and use of laboratory animals” prepared by the National Academy of Sciences and published by the National Institutes of Health [17].

### 2.3. Extraction of Plant Samples

50 g of the powder of the aerial part of *Thymus leptobotrys* is placed in a cartridge and placed in the Soxhlet extractor [18]. The flask is filled with 300 ml methanol for the methanolic extract and 300 ml ethanol for the ethanolic extract, and the flask is placed on a heater. When heated, the solvent evaporates, condenses in the cooler, falls back into the Soxhlet extractor, solubilizes the active ingredients, and returns to the recovery flask: the operation is repeated several times for 6 hours until the powder is completely depleted. The extract solution is cooled to room temperature, filtered through a filter paper, and then freed of all traces of solvent by means of a rotary vacuum evaporator at 40°C. The extracts are stored in hermetically sealed brown glass vials at 4°C. The extract yield (%) is calculated as follows:

(1) $$\begin{array}{c} \text{yield}\%=\frac{\text{extract}\left(\text{g}\right)}{\text{dry matter}\left(\text{g}\right)}\times 100. \end{array}$$

### 2.4. Determination of Phenolic Content

#### 2.4.1. Total Phenol Content

The total content of polyphenol is determined by the Folin–Ciocalteu reagent protocol as described by Nounah et al. [19]. First, 0.5 mL of the extract is mixed with 2.5 mL of Folin–Ciocalteu reagent diluted 1/10 with distilled water. Then, 4 mL of 7.5% Na_2_CO_3_ (w/v) is added, followed immediately by incubation for 30 min at 45°C. The spectrophotometric reading at 760 nm is taken against a blank. The calibration curve is performed with gallic acid at concentrations between 2.5 and 250 *μ*g/mL. The total content of phenols is expressed in mg of gallic acid equivalent (GAE) per gram of extract (mg GAE/g E).

#### 2.4.2. Total Flavonoid Content

The total content of flavonoids is determined using the aluminum chloride colorimetric method [19]. 0.25 mL of the extract is placed in a test tube containing 1.25 mL of distilled water, followed by the addition of 0.075 mL of sodium nitrite solution NaNO_3_ (5%), and the mixture is kept at rest for 5 minutes. Then 0.15 mL of 10% aluminum chloride is added. After 6 min, 0.5 mL 1M sodium hydroxide is added. The mixture is diluted with 0.275 mL of distilled water. The final mixture is then incubated for 30 minutes in the dark at room temperature, and the absorbance is measured at 510 nm. The flavonoid content is expressed in mg of rutin equivalent (RE) per gram of extract (mg RE/g E).

#### 2.4.3. Total Tannin Content

The total content of condensed tannins is determined by the acidic vanillin method [20]. 500 *μ*L of extract solution is mixed with 3 mL of 4% vanillin-methanol solution and 1.5 mL hydrochloric acid. The mixture is allowed to stand in the dark for 15 min. The absorbance is measured at 500 nm. The calibration curve for catechin is constructed from the concentrations from 30 to 1000 *μ*g/mL. The content of condensed tannins is expressed in mg of catechin equivalent (CE) per gram of extract (mg CE/g E).

### 2.5. Antioxidant Activity

#### 2.5.1. DPPH Free Radical Scavenging

The free radical scavenging activity of the extracts was measured by DPPH using the method described by Re et al. [21]. A 0.2 mM solution of DPPH is prepared in methanol or ethanol, and 0.5 mL of this solution is added to 2.5 mL of the sample. After vigorous shaking, the mixture is kept in the dark for 30 min. The absorbance is measured at 517 nm. Trolox is used as a reference compound. The experiment is performed in triplicate. The ability to recover the DPPH radical is measured using the following equation:

(2) $$\begin{array}{c} \%\text{inhibition}=\frac{A_{0}-A_{1}}{A_{0}}\times 100, \end{array}$$

where *A*_0_ is the absorbance of the negative control and *A*_1_ is the absorbance of the sample.

The scavenging activity is expressed by the IC50 which represents the sample concentration required to inhibit 50% of the free radical scavenging activity.

#### 2.5.2. Trolox Equivalent Antioxidant Capacity (TEAC)

The ABTS radical scavenging activity is determined by the protocol described by Nounah et al. [22]. Stock solutions of 7 mM ABTS and 2.4 mM potassium persulfate (K_2_S_2_O_8_) in identical volumes are kept in the dark for sixteen hours at room temperature. Prior to testing, the ABTS+ solution is diluted in methanol or ethanol to give an absorbance of 0.700 ± 0.02 at 734 nm. 2 mL of the resulting solutions is allowed to react with 200 *μ*L of the sample (2 mg/mL), and the absorbance is measured after 30 min at 734 nm. The same procedure is used with Trolox at different concentrations (from 5 to 100 *μ*g/mL). The percentage of ABTS^**+**^ inhibition by different concentrations is calculated, and the antioxidant power of the sample is represented in Trolox equivalent (mg TE/g sample). The test is performed in triplicate.

### 2.6. Acute Toxicity Study

The acute toxicity study of *T. leptobotrys* extracts is evaluated on adult rats in accordance with the Organization for Economic Cooperation and Development 423 guidelines [23, 24]. After a fasting period of 3-4 h, the body weight of each animal is measured to determine the dose to be administered orally, expressed as mg extract per kg body weight. The animals are arbitrarily divided into three groups of six rats (*n* = 6). The first and second groups receive methanolic and ethanolic extracts from *T. leptobotrys* at doses of 300 and 2000 mg/kg, and the third group (control group) is given distilled water (control vehicle) orally. Signs of toxicity evaluated are general behavioral symptoms, changes in body weight, ingestion of water and food, respiration, convulsions, and mortality. They are assessed systematically for each group during the first few hours and then 14 days after treatment. The 50% lethal dose (LD50) is determined according to the protocol described in guidelines 423 [23].

### 2.7. Anti-Inflammatory Effect

The anti-inflammatory effect is studied using the carrageenan-induced paw edema method [25, 26]. Wistar rats are divided into four groups (*n* = 6). The animals were fasted for 18 hours prior to testing. The groups of rats were given different oral concentrations of *T. leptobotrys* extracts (300 and 600 mg/kg). The control group receives distilled water while the last group receives indomethacin (10 mg/kg) as the reference drug. After 30 minutes, all rats are injected subcutaneously with carrageenan solution (0.05 mL of 1% carrageenan suspended in 0.9% NaCl) into the subplantar region of the left hind paw. The paw volumes of the rats were recorded with a LE7500 plethysmometer just before the carrageenan injection (*V*_0_), then at 1 h 30 min, 3 h, and 6 h after the carrageenan injection (*V*_*t*_). Anti-inflammatory effect is calculated using the following equation [27]:

(3) $$\begin{array}{c} \%\text{inhibition}=\frac{\left(V_{t}-V_{0}\right)\text{ control}-\left(V_{t}-V_{0}\right)\text{ treated group}}{\left(V_{t}-V_{0}\right)\text{ control}}\times 100. \end{array}$$

### 2.8. Statistical Analysis

The data are expressed as mean values ± standard deviation for each measurement and analyzed by means of analysis of variance (one-way ANOVA) followed by Tukey posttests. The statistical study is performed using GraphPad Prism 8 software. A probability of *P* < 0.05 indicates that the values are considered statistically significant.

## 3. Results and Discussion

### 3.1. Extraction Yield

The yield of the methanolic extract is around 22.2%, higher than that of ethanolic extract (15.8%).

### 3.2. Determination of Phenolic Content

Table 1 shows the total content of phenolics (TCP), flavonoids (TCF), and condensed tannins (CCT) of methanolic and ethanol extracts of *T. leptobotrys*. The highest TCP, TCF, and CCT are found in the methanolic extract. The ethanolic extract, on the other hand, has slightly lower levels of TCP and TCF, while the content of CCT is extremely low. This variation in results clearly shows that the difference in polarity of the solvents influences the extraction of phenolic compounds [28].

### 3.3. Antioxidant Activity

The antioxidant capacity of methanolic and ethanol extracts of *T. leptobotrys* was studied using two different methods: DPPH and ABTS radical absorption capacity (Table 2). A low IC50 value indicates significant antioxidant activity. According to the DPPH method, both types of extracts have significant antioxidant activity (Table 2), especially the methanolic extract (IC50 = 12.363 ± 0.324 *μ*g/mL), compared to the ethanolic extract (IC50 = 20.693 ± 0.182 *μ*g/mL). In both cases, the IC50s are higher than that of the Trolox standard (IC50 = 1.810 ± 0.017 *μ*g/mL). The antioxidant activity of *T. leptobotrys* extracts, according to the ABTS test, shows that the methanolic extract has a powerful antioxidant activity of the order of 930.935 ± 1.513 mg TE/g extract, higher than that found in the ethanolic extract (860.309 ± 0.954 mg TE/g).

The methanolic extract of the aerial part of *T. leptobotrys* has a higher DPPH antioxidant activity than both the methanolic extract of the leaves of *T. leptobotrys* (IC50 = 1950 *μ*g/mL) and the methanolic extract of the stems of *T. leptobotrys* (IC50 = 430 *μ*g/mL) using the DPPH test [29]. Thus, the whole aerial part of the plant is logically more active than individual leaves or stems.

Comparison of the results with the other studies is however not appropriate for the following two reasons: on the one hand, the antioxidant content is strongly influenced by the type of solvent used, and on the other hand, the results are expressed in caffeine equivalents or Trolox equivalents, which makes the results not directly comparable [30].

The important antioxidant activity of the methanolic extract of *Thymus leptobotrys* to the DPPH and ABTS radical could be explained by its richness in phenolic compounds. There is indeed a correlation between the antioxidant activity and content of phenolic compounds [31–33]. On the other hand, many studies have reported that phenolic compounds are often known by their antioxidant activity [34–36].

### 3.4. Acute Toxicity Study

The acute toxicity study of methanol and ethanol extracts of *T. leptobotrys* showed no mortality or clinical signs of toxicity in each group of animals all through the fourteen days of study. At 2000 mg/kg, rats showed no signs of changes in behavioral patterns or undesirable pathology or weight loss (Table 3). Ethanol and methanol extracts of *T. leptobotrys* can be classified as category 5 and are considered to be nontoxic by the oral route [23].

### 3.5. Anti-Inflammatory Effect

**Figure 1:**
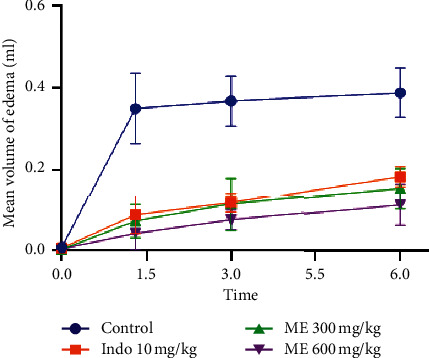
Influence of ME: methanolic extract of *Thymus leptobotrys* (300 and 600 mg/kg) on carrageenan edema. The data represent the difference in the mean volume of the edema (mean ± SEM), *n* = 6. *P* ≤ 0.001 versus witness.

**Figure 2:**
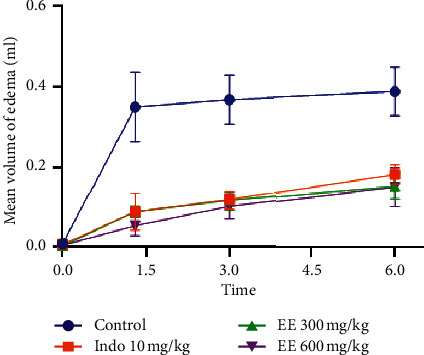
Influence of EE: ethanolic extract of *Thymus leptobotrys* (300 and 600 mg/kg) on carrageenan edema. The data represent the difference in the mean volume of the edema (mean ± SEM), *n* = 6. *P* ≤ 0.001 versus witness.

The anti-inflammatory effect of ethanolic and methanolic extracts is evaluated by the carrageenan-induced rat paw edema method. The results are presented in Table 4 and Figures 1 and 2. Six hours after administration of *T. leptobotrys* at 600 mg/kg extract, the volume of edema decreased notably compared to the control group (*P* < 0.001) (Figures 1 and 2). Rats treated with methanol and ethanol extracts showed the greatest decrease in inflammation (90.04% and 83.88%, respectively) after 1.5 h of carrageenan injection. This reduction in edema was greater than that of indomethacin 76.77% (Table 4) and was maintained throughout the observation period. Based on these results, it can be concluded that *T. leptobotrys* extracts act within the first hour on the initial phase of inflammation, just like indomethacin.

Carrageenan-induced paw edema is due to cyclooxygenase and lipoxygenase. The cyclooxygenase enzyme is directly involved in inflammation through prostaglandin production while lipoxygenase indirectly causes an inflammatory response [7, 37]. Thus, the anti-inflammatory power of methanolic and ethanol extracts of *T. leptobotrys* can be explained by an inhibitory action exerted on cyclooxygenases. The inhibition of cyclooxygenases may be due to the richness of methanolic and ethanolic extracts in polyphenolic constituents. Polyphenols prevent the formation of prostaglandins that cause inflammation [38]. Tannins and flavonoids also contribute to the anti-inflammatory effect through their ability to inhibit the production of 5 proinflammatory mediators such as lipoxygenase, prostaglandins, cyclooxygenase, serotonin, histamines, and cytokines such as IL-8, TNF-*α*, or IL-1*β* [39–41].

## 4. Conclusion

The methanolic and ethanolic extracts' chemical analysis of the aerial part of *Thymus leptobotrys* reveals their phenolic compounds abundance. Overall, the methanolic extract has the highest levels of phenolic compounds as well as a more pronounced antioxidant activity using both DPPH and ABTS methods, with values of IC50 = 12.363 ± 0.324 *μ*g/mL and 930.935 ± 1.513 *μ*g extract TE/g, respectively. The decrease in leg edema in adult rats after 6 hours of administrating 600 mg extract/kg shows a strong anti-inflammatory effect for both extracts. The toxicity test indicates an LD50 above 2000 mg/kg for both extracts. These results show the presence of bioactive molecules in the aerial fraction of *Thymus leptobotrys*. Further studies for future use against diseases due to oxidative stress are recommended.

## Figures and Tables

**Table 1:** Total content of phenols (TCP), flavonoids (TCF), and condensed tannins (CCT) of ethanolic and methanolic extracts of *Thymus leptobotrys*.

Extracts	TCP (mg GAE/g E)	TCF (mg RE/g E)	CCT (mg CE/g E)
Methanol extract	243.08 ± 2.911	179.28 ± 0.922	39.31 ± 0.441
Ethanol extract	214.26 ± 2.079	144.41 ± 1.537	3.06 ± 0.200

**Table 2:** Antioxidant activity of ethanolic and methanolic extracts of *Thymus leptobotrys*.

Extracts	DPPH (IC50 *μ*g/mL)	ABTS (mg TE/g extract)
Methanol extract	12.363 ± 0.324	930.935 ± 1.513
Ethanol extract	20.693 ± 0.182	860.309 ± 0.954
Trolox	1.850 ± 0.017	—

**Table 3:** Study of the acute toxicity of ethanolic and methanolic extracts of *Thymus leptobotrys* administered by gavage to rats.

Treatments	Dose mg/kg	Mortality	Toxic symptoms	Changes in body weight (g)		
				1^st^ day	14^th^ day	Difference
Methanol extract	2000	—	None	185.03 ± 1.02	197.83 ± 2.62	+12.8
Ethanol extract	2000	—	None	185.63 ± 2.42	197.86 ± 1.37	+12.23
Control	—	—	—	186.03 ± 1.77	199.06 ± 0.68	+13.03

**Table 4:** Percentage inhibition of left hind leg volume in rats treated with *Thymus leptobotrys* ethanolic and methanolic extracts.

Treatments (mg/kg)	Inhibition of edema induced by carrageenan (%)		
	1 h 30 min	3 h	6 h
Indomethacin, 10	76.77	75.78	61.37
Methanol extract, 300	65.87	64.12	57.51
Methanol extract, 600	90.04	84.30	75.10
Ethanol extract, 300	68.24	61.43	55.36
Ethanol extract, 600	83.88	76.68	62.66

## Data Availability

All data supporting the findings are adequately included within the article.
